# Inhibition of human lung cancer cell proliferation and survival by wine

**DOI:** 10.1186/1475-2867-14-6

**Published:** 2014-01-23

**Authors:** Carly C Barron, Jessy Moore, Theodoros Tsakiridis, Gary Pickering, Evangelia Tsiani

**Affiliations:** 1 Department of Health Sciences, Brock University, St. Catharines, Ontario L2S 3A1, Canada; 2 Department of Oncology, McMaster University, Hamilton, Ontario L8S 4L8, Canada; 3 Department of Biological Sciences, Cool Climate Oenology and Viticulture Institute (CCOVI), Brock University, St. Catharines, Ontario L2S 3A1, Canada

**Keywords:** Cancer, Proliferation, Survival, Akt, Erk, p53, Wine

## Abstract

**Background:**

Compounds of plant origin and food components have attracted scientific attention for use as agents for cancer prevention and treatment. Wine contains polyphenols that were shown to have anti-cancer and other health benefits. The survival pathways of Akt and extracellular signal-regulated kinase (Erk), and the tumor suppressor p53 are key modulators of cancer cell growth and survival. In this study, we examined the effects of wine on proliferation and survival of human Non-small cell lung cancer (NSCLC) cells and its effects on signaling events.

**Methods:**

Human NSCLC adenocarcinoma A549 and H1299 cells were used. Cell proliferation was assessed by thymidine incorporation. Clonogenic assays were used to assess cell survival. Immunoblotting was used to examine total and phosphorylated levels of Akt, Erk and p53.

**Results:**

In A549 cells red wine inhibited cell proliferation and reduced clonogenic survival at doses as low as 0.02%. Red wine significantly reduced basal and EGF-stimulated Akt and Erk phosphorylation while it increased the levels of total and phosphorylated p53 (Ser15). Control experiments indicated that the anti-proliferative effects of wine were not mediated by the associated contents of ethanol or the polyphenol resveratrol and were independent of glucose transport into cancer cells. White wine also inhibited clonogenic survival, albeit at a higher doses (0.5-2%), and reduced Akt phosphorylation. The effects of both red and white wine on Akt phosphorylation were also verified in H1299 cells.

**Conclusions:**

Red wine inhibits proliferation of lung cancer cells and blocks clonogenic survival at low concentrations. This is associated with inhibition of basal and EGF-stimulated Akt and Erk signals and enhancement of total and phosphorylated levels of p53. White wine mediates similar effects albeit at higher concentrations. Our data suggest that wine may have considerable anti-tumour and chemoprevention properties in lung cancer and deserves further systematic investigation in animal models of lung cancer.

## Introduction

Cancer cells are characterized by accelerated proliferative capacity and resistance to apoptosis (programmed cell death). Non-small cell lung cancer (NSCLC) accounts for 85% of lung cancer cases and despite utilization of aggressive radio- and/or chemotherapy, fewer than 20% of such patients reach a 5 year survival [1]. This is due to significant resistance of NSCLC to such conventional cytotoxic therapies. Therefore, there is an urgent need to identify new effective prevention strategies and therapies for NSCLC.

Molecular signaling pathways of growth factor receptors, such as the Epidermal Growth Factor (EGF) Receptor (EGFR), stimulate cancer cell growth, survival and resistance to cytotoxic therapy. Binding of EGF activates EGFR and initiates signal transduction pathways, including the phosphatidylinositol 3-kinase (PI3K)-Akt and Ras-Erk, resulting in cell proliferation and survival [2]. Increased EGFR signaling is linked to cancer and abnormal EGFR expression is found in approximately 80% of cases of NSCLC [2]. Although EGFR tyrosine kinase inhibitors such as gefitinib and erlotinib provide a therapeutic benefit in treating patients with NSCLC, particularly those with EGFR mutations, most patients eventually develop resistance to such targeted therapies [2,3].

Activation of PI3K by EGFR generates 3-phosphoinositides which bind and activate phosphoinositide-dependent kinase-1(PDK-1). PDK-1 phosphorylates and activates the serine threonine kinase Akt (protein kinase B (PKB)) [4,5]. Activated Akt in turn phosphorylates tuberous sclerosis complex 2 (TSC2) leading to activation of the mammalian target of rapamycin (mTOR) and p70 S6 kinase, resulting in stimulation of protein synthesis, growth and proliferation [4,5]. Activated Akt also phosphorylates Bad, a proapoptotic Bcl-2 family member, causing its degradation resulting in inhibition of apoptosis [4,5]. Akt is a proto-oncogene and activated Akt leads to cancer cell proliferation, survival, and resistance to chemo and radio-therapy [4-6]. Several studies have shown that Akt is overexpressed in various cancer cell lines and human malignancies [5,6]. Increased Akt phosphorylation, a marker of activation of this molecule [4], is seen in many cancer cells [5-8]. On the other hand, targeting/inhibiting the Akt signaling pathway inhibits cancer cell growth and enhances apoptosis [5,6].

The extracellular signal-regulated kinase (Erk) is a member of the mitogen-activated protein kinase (MAPK) family, shown to be up-regulated and involved in cell proliferation and survival in various cancer cells [9].

The tumour suppressor p53 pathway is frequently inactivated in cancer cells and both chemotherapy and radiotherapy resistance has been strongly associated with reduced p53 function [10]. Cytotoxic anti-cancer therapies induce DNA-damage and/or -stress and activate signaling cascades including the p53 pathway. This leads to inhibition of cancer cell proliferation and induction of apoptosis. In response to stress/DNA damage, p53 is phosphorylated on multiple serine residues, including Ser 15, resulting in inhibition of degradation and increased p53 levels. Phosphorylated/activated p53 binds to downstream targets leading to cell cycle arrest and/or apoptosis, both detrimental to the uncontrolled growth of cancer cells [10].

Moderate consumption of red wine has been established to have cardiovascular benefits [11]. A limited number of *in vitro* studies using cancer cells [12-14], and some epidemiological studies [11], indicate that red wine possesses anti-cancer properties. Red wine is a rich source of the polyphenol resveratrol which has been shown to inhibit cancer cell proliferation, induce apoptosis [15,16], and in a recent study by our group found to sensitize prostate cancer cells to radiotherapy [17]. Although many studies examined the effects of resveratrol on cancer cell proliferation, investigations into the effect of whole wine are limited. Interestingly, high intake of beer or spirits is associated with increased risk of lung cancer, while red wine intake is associated with a reduced risk [18,19]. Studies indicate that regular and moderate red wine consumption could have beneficial health effects [20] and is associated with a 22% decrease in risk of cancer [21].

*In vitro* studies have shown inhibition of growth of human oral squamous carcinoma (SCC-25) cells [12], and human prostate cancer cells [14] by red wine. In addition, studies using red wine polyphenol extracts found inhibition of breast cancer [22], colon carcinoma [23], and skin epidermal [24] cells.

In the present study we found a significant inhibition of human lung cancer cell proliferation and survival, inhibition of Akt and Erk and induction of p53 by wine.

## Results

### Red wine inhibits proliferation and survival of A549 lung cancer cells

**Figure 1:**
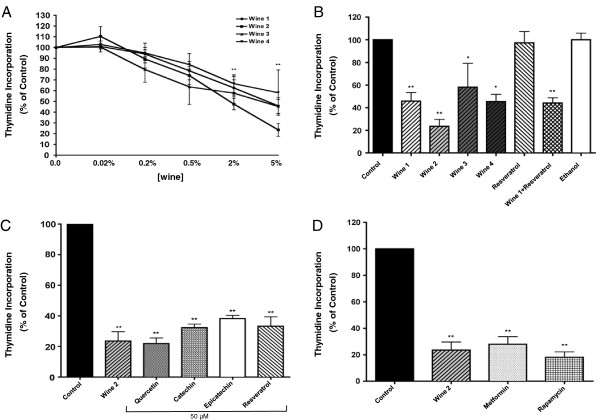
**Effect of red wine on thymidine incorporation in A549 NSCLC cells.** A549 cells were deprived of serum for 24 h then incubated with **(A)** the indicated wine concentrations, **(B)** without (control) or with 5% wine or 1.1 μM resveratrol or ethanol (to match the ethanol concentration in the cells exposed to 5% wine), **(C)** without (control) or with 5% wine or 50 μM of quercetin, catechin, epicatechin or resveratrol, **(D)** without (control) or with 5% wine, 1 mM metformin or 25 nM rapamycin for 24 h at 37°C. Cells were washed and [^3^H]thymidine incorporation was measured as indicated in the Methods. The results are the mean ± SE of 4 independent experiments performed in triplicate and expressed as percent of control. *P < 0.05, **P < 0.01 vs. control.

The physicochemical composition of the four red wines used in this study (W1-4) is shown in Table 1. There were no significant differences in the basic physicochemical composition of the wines with the exception that the phenolic content of Pinot Noir (W2) was higher than that of the other wines used. Sub-confluent, 24 hour serum-deprived A549 lung cancer cells were incubated in media containing 0.02, 0.2, 0.5, 2, or 5% red wine for 24 hours. All red wines used in the study inhibited thymidine incorporation into cells in a dose-dependent manner (Figure 1A).

**Table 1:** Basic physicochemical composition and phenolic content of wines

**Wine**	**Viscosity**	**Residual sugar**	**Titratable acidity**	**Ethanol**	**Glycerol**	**pH**	**Total phenolics**
	**(centistrokes/min)**	**(g/l)**	**(g/l)**	**(%v/v)**	**(g/l)**		**(mg/L gallic acid equivalents)**
Cabernet Sauvignon	1.68	2.75	7.37	11.36	7.58	3.26	1868.3
Late harvest 2001 (W1)							
Pinot Noir	1.65	4.12	7.02	11.91	5.54	3.43	2681.3*
2001 (W2)							
Cabernet Franc	1.69	3.62	8.30	12.81	6.64	3.16	1378.8
2001 (W3)							
Cabernet Sauvignon	1.58	5.75	8.17	10.28	5.35	3.15	1641.3
Early harvest 2001 (W4)							

Data represent the average values of duplicate measurements of wine samples. *P < 0.05.

Red wine contains the polyphenol resveratrol which is known to have anti-proliferative effects [16,25]. The resveratrol levels of the wines used in our study were not measured but based on the literature [11] the average concentration of resveratrol in red wine ranges from 1-5 mg/L. Assuming that the resveratrol (MW:228.24 g/mol) concentration in our wine was 5 mg/L it is calculated that at the highest wine concentration (5%) used in our study, the cells would have been exposed to 1.1 μM resveratrol and therefore we used this concentration of resveratrol and examined cell proliferation. Figure 1B shows that A549 cell proliferation was significantly inhibited by 5% red wine (W1: 45.85 ± 2.44, W2: 23.54 ± 6.09, W3: 58.10 ± 21.11, and W4: 45.32 ± 6.54% of untreated control) while no significant effect on cell proliferation were seen with 1.1 μM resveratrol (97.33 ± 9.97% of control). Interestingly, the combination of 1.1 μM resveratrol and wine (44.06 ± 4.65% of control) inhibited cell proliferation to the same level as wine alone. The ethanol content of the wines used in our study was between 10.28 and 12.81% with an average of 11.59% (Table 1). Thymidine incorporation in cells treated with ethanol, to match the ethanol in 5% wine-treated cells, was not different than control cells (100.03 ± 1.73% of control) (Figure 1B). Apart from resveratrol, red wine contains other polyphenols including quercetin, catechin and epicatechin all of which have been shown to inhibit cancer cell proliferation at concentrations ranging from 50–100 μM [26]. Comparing the effect of red wine with that of 50 μM of each polyphenol we saw inhibition of cancer cell proliferation by red wine (23.54 ± 6.09) to levels similar with that achieved with 50 μM of each polyphenol (21.9 ± 3.7, 32.3 ± 2.35, 38.23 ± 2.13, 33.26 ± 6.11% of control for quercetin, catechin, epicatechin and resveratrol, respectively) (Figure 1C).

We also compared the effect of red wine with that of the mTOR inhibitor rapamycin [5,27] and the anti-diabetic drug metformin [28], both of which inhibit signaling events downstream of PI3K and are under investigation as promising therapeutics in cancer. Exposure of A549 cells to 1 mM metformin or 25 nM rapamycin resulted in a significant inhibition of cell proliferation (28 ±5.7, 18.1 ± 4.1% of control for metformin and rapamycin, respectively) that was at a similar level to that achieved with red wine (Figure 1D).

**Figure 2:**
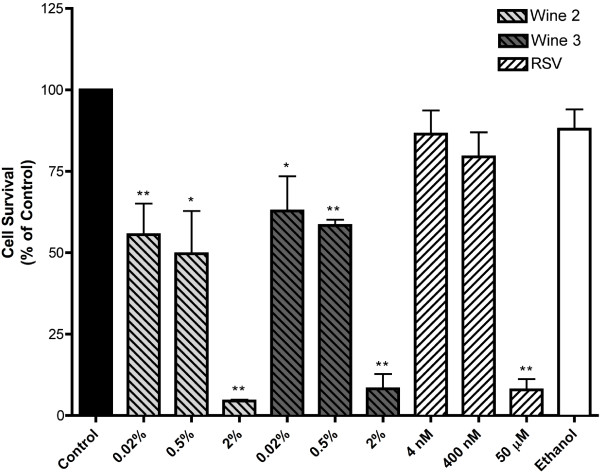
**Effect of red wine on survival of A549 cells.** Cells were incubated with the indicated concentrations of red wine, resveratrol or ethanol (to match the ethanol in 2% wine-treated cells) for 7 days followed by fixing and staining with 0.05% methylene blue and colonies (> 50 cells) were counted. Results are expressed as the surviving fraction compared to untreated control. RSV = Resveratrol, *P < 0.05, **P < 0.01 vs. control.

We next examined the effect of red wine on clonogenic cell survival. Exposure of the cells to 0.02 and 0.5% of two red wines (Wine 2 and 3) resulted in a significant inhibition of clonogenic cell survival while 2% red wine abolished most of the ability of cells to create surviving colonies (residual colony formation was W2: 4.5 ±0.38, W3: 8.21 ± 5.8% of control) (Figure 2). Exposure of the cells to 4 nM and 400 nM resveratrol, to match the resveratrol levels in cells exposed to 0.02% and 2% wine, respectively, caused minor, non-significant, inhibition of cell survival but exposure to 50 μM resveratrol did inhibit clonogenic survival (7.8 ± 3.29) to levels close to those achieved by 2% of wines 2 and 3 (Figure 2). Similarly, exposure of the cells to ethanol, to match the ethanol levels in cells treated with 2% wine, did not have a significant effect on cell survival (Figure 2). These results indicate that red wine induces a significant inhibition of lung cancer cell survival that cannot be explained by either the content of resveratrol or ethanol in the wine.

### Effect of red wine on glucose transport

**Figure 3:**
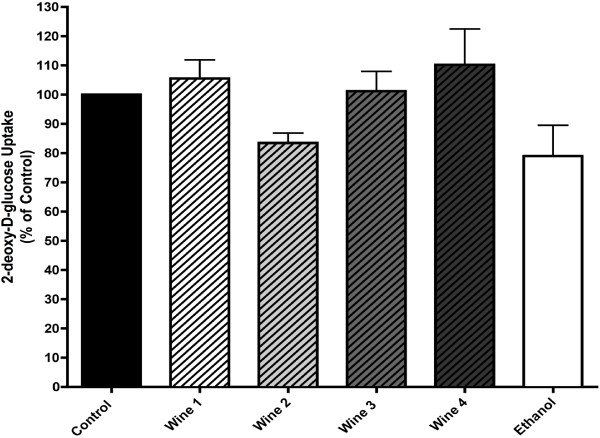
**Effect of red wine on glucose uptake.** Serum-deprived A549 cells (24 h) were treated without (control) or with 5% red wine, or ethanol vehicle control for an additional 24 h at 37°C. Cells were washed and [^3^H]-2 deoxy-glucose uptake was measured as discussed in the Methods. The results are the mean ± SE of 5 independent experiments performed in triplicate and expressed as a percent of control.

Resveratrol has been suggested to directly inhibit glucose uptake in cancer cells [29] and it has been previously shown that caloric restriction using a non-metabolizable glucose analogue inhibits cell proliferation [30]. Therefore, it was possible that the observed effects of red wine in the present study were due to inhibition of glucose uptake. For that, we examined the effect of wine on glucose uptake in A549 cells. None of the red wines used in this study significantly affected glucose uptake by A549 cells (W1: 105.52 ± 6.35, W2: 83.47 ± 3.41, W3: 101.16 ± 6.81, W4: 110.21 ± 12.29% of control) (Figure 3) suggesting that the ability of red wine to inhibit cancer cell proliferation is not dependant on modulation of glucose uptake.

### Red wine inhibits Akt and Erk phosphorylation/activation

**Figure 4:**
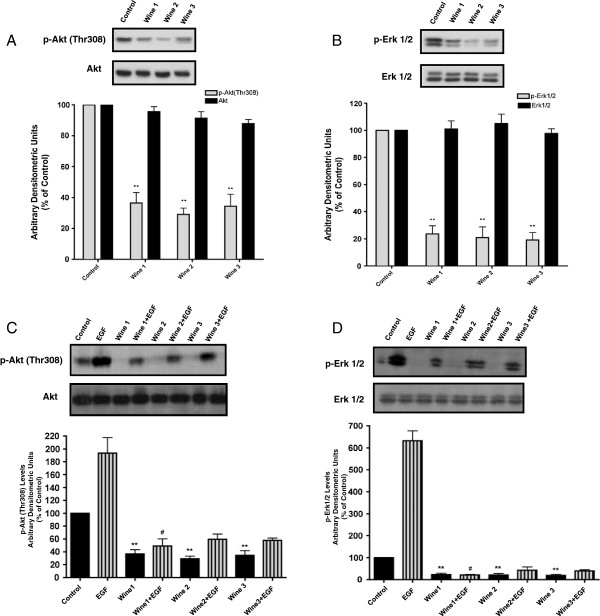
**Effect of red wine on Akt and ERK.** Total cell lysates were prepared from A549 cells that were serum-deprived for 24 h then treated with or without 5% wine for 24 h. Stimulation with 100 ng/ml EGF was performed for 10 min before cell lysis **(C, D)**. Cell lysates (20 μg) were resolved by SDS-PAGE and immunoblotted with anti-phospho, or anti-total- Akt antibody **(A, C)** or anti-phospho, or anti-total- Erk antibody **(B, D)**. Representative immunoblots of 3 independent experiments are shown. Immunoblots were scanned to quantitate the density of the bands and the results in the bar graphs are the mean ± SE of 3 independent experiments. All values are arbitrary densitometry units expressed relative to untreated control. *P < 0.05, **P < 0.01 vs. control, #p < 0.05 vs. EGF.

Untreated A549 cells were found to have high levels of Akt Thr308 phosphorylation (which mediates the activation of this enzyme) and treatment with red wine was found to significantly inhibit this elevated basal Akt phosphorylation. Wines 1–3 were used in these experiments and all induced greater than 60% inhibition of Akt Thr308 phosphorylation (W1: 36.55 ± 6.7%, W2: 29.16 ± 4.07%, W3: 34.41 ± 7.59% of control) (Figure 4A) without significantly affecting the total levels of Akt (W1: 95.67 ± 3.18%, W2: 91.4 ± 4.28%, W3: 87.90 ± 2.67% of control, Figure 4A).

Similarly, untreated A549 cells showed high levels of basal Erk1/2 phosphorylation and red wine (Wines 1–3) caused a marked inhibition of this activity reducing basal Erk phosphorylation by more than 75% (W1:23.66 ± 5.9%, W2:20.94 ± 7.8%, W3: 19.20 ± 5.4%, of control, Figure 4B) without affecting the total levels of this enzyme (W1:101.01 ± 5.99, W2:105.05 ±7.00, W3: 97.7 ± 3.57%, of control, Figure 4B).

We also examined the effect of red wine on EGF-stimulated Akt and Erk1/2 phosphorylation. Exposure of the cells to 100 ng/ml EGF caused a rapid (within 10 min) and significant enhancement of Akt phosphorylation (EGF: 193.46 ± 23.98 compared to control, Figure 4C). Importantly, red wine (Wines 1–3) abolished both the basal and the EGF-stimulated Akt phosphorylation (W1+ EGF: 49.01 ± 11.32, W2+ EGF: 59.43 ± 8.1, W3+ EGF: 57.89 ± 3.43% of control, Figure 4C). Similarly, EGF induced a greater than 5-fold increase in Erk phosphorylation and red wine (1–3) abolished both basal and EGF-stimulated Erk phosphorylation (EGF: 538 ± 97, W1 + EGF: 25.16 ± 4.29, W2 + EGF: 31.98 ± 13.86, W3 + EGF: 28.06 ± 12.36% of control, Figure 4D).

### Red wine increases p53 levels

**Figure 5:**
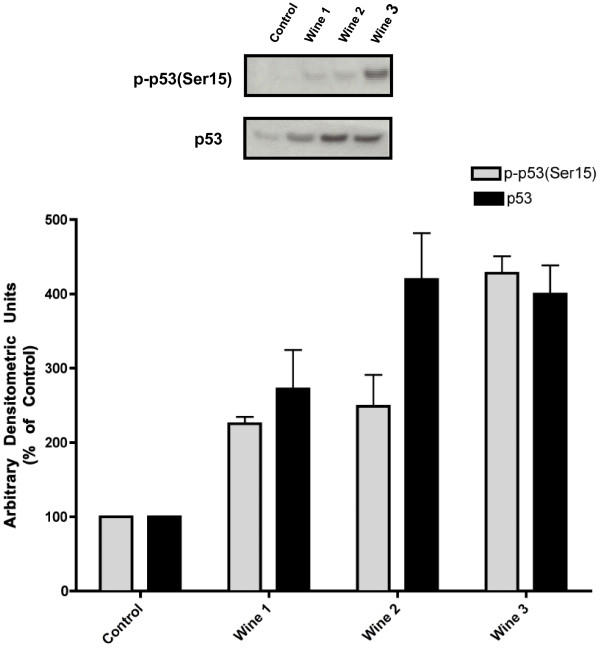
**Effect of red wine on p53.** Serum-deprived (24 h) A549 cells were treated with or without 5% red wine for 24 h. Total cell lysates were prepared and 20 μg of protein from each sample were resolved by SDS-PAGE and immunoblotted with anti-phospho, or anti-total- p53 antibody. A representative immunoblot is shown. Immunoblots were scanned to quantitate the density of the bands and the results in the bar graphs are the mean ± SE of 3 independent experiments. All values are arbitrary densitometry units expressed relative to untreated control.

Red wine increased total p53 levels by 2–4 fold (W1: 272.07 ± 52.5, W2: 419.3 ± 62.51, W3:399.72 ± 38.56% of control, Figure 5). Similarly, the levels of Ser15 p53 phosphorylation were increased by 2–4 fold (W1: 206 ± 34, W2: 283 ± 99, W3:467 ± 32% of control) in response to red wine treatment (Figure 5).

### White wine inhibits survival of A549 cells and reduces Akt phosphorylation levels

**Figure 6:**
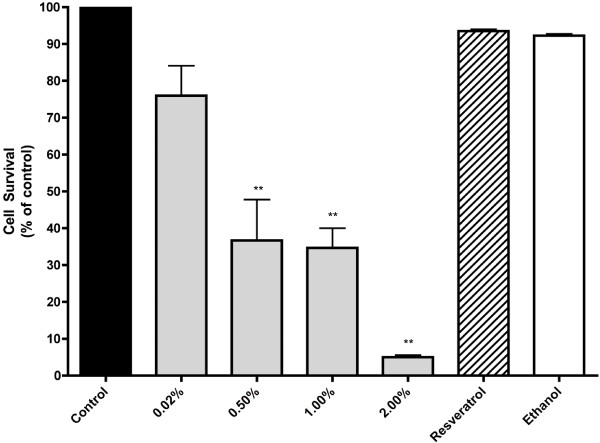
**Effects of white wine on clonogenic survival of A549 cells.** Cells were incubated with the indicated concentrations of white wine (Riesling), resveratrol or ethanol (to match the concentrations found in 2% white wine-treated cells) for 7 days followed by fixing and staining with 0.05% methylene blue and colonies (>50 cells) were counted. Results are expressed as the surviving fraction compared to untreated control of 3–5 individual experiments. RSV = resveratrol, **P < 0.01 vs. control.

To examine whether the anti-proliferative and signaling effects of wine were specific to red wine, we examined the effects of the white wine Riesling (produced in CCOVI, 2013). Total phenolic content of Riesling was measured and found to be (3.41 mg/L gallic acid equivalents) which is lower than the concentrations found in the red wines. The other basic physicochemical composition of white wine including pH and ethanol content were not different from the red wines used. Exposure of cells to 0.02, 0.5, 1 and 2% white wine also resulted in a dose-dependent inhibition of cell survival (Figure 6). A significant inhibition of cell survival was seen with 0.5% white wine, while at 2%, the inhibition of clonogenic survival (5% of control) was comparable to that seen with red wine. White wine contains lower levels of resveratrol (0.5-0.8 mg/ml) compared to red wine. When cells were exposed to 3.24 nM resveratrol to match the resveratrol levels in cells exposed to 2% white wine, cell survival was not affected. Similar results were observed when cells were exposed to ethanol to match the ethanol in cells treated with 2% white wine (Figure 6).

**Figure 7:**
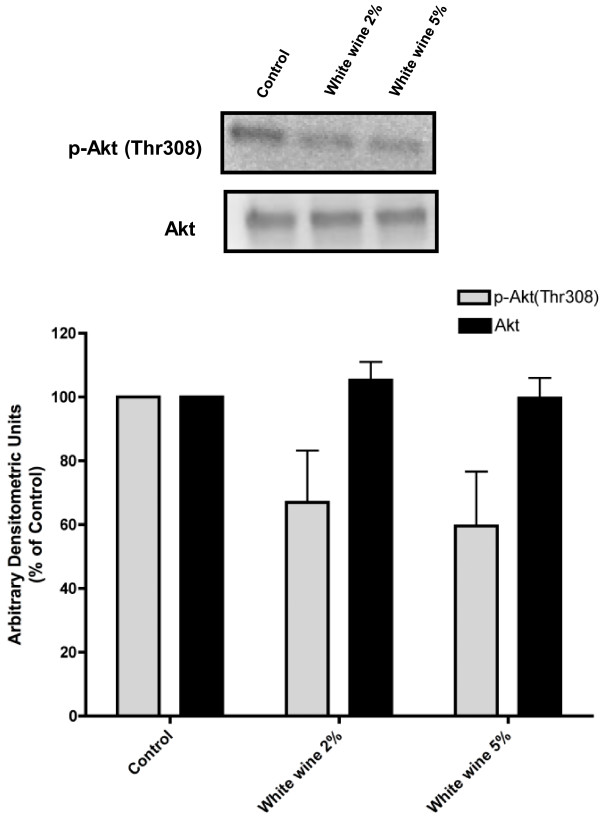
**Effect of white wine on Akt in A549 NSCLC cells.** Total cell lysates were prepared from A549 cells that were serum-deprived for 24 h then treated with or without 2% or 5% white wine (Riesling) for 24 h. Cell lysates (25 μg) were resolved by SDS-PAGE and immunoblotted with anti-phospho or anti-total- Akt antibody. Representative immunoblots of 2 independent experiments are shown. Immunoblots were scanned to quantitate the density of the bands and the results in the bar graphs are mean ± SE. All values are arbitrary densitometry units expressed relative to untreated control.

To examine the effects of white wine on cellular signaling events, we analyzed its effects on Akt phosphorylation. Exposure of A549 cells to 2% and 5% white wine resulted in inhibition of basal Akt phosphorylation (67% and 59% of control respectively), while total Akt levels were not affected (Figure 7).

### Inhibition of Akt phosphorylation by wine in H1299 cells

**Figure 8:**
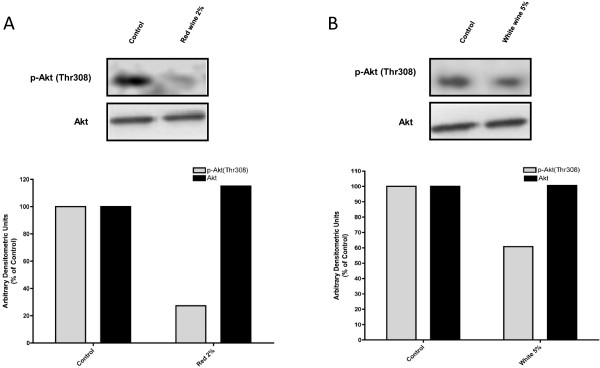
**Effect of wine on Akt in H1299 NSCLC cells.** Total cell lysates were prepared from H1299 cells that were serum-deprived for 24 h then treated with or without 2% red wine (Pinot Noir) **(A)** or 5% white wine (Riesling) **(B)** for 24 h. Cell lysates (25 μg) were resolved by SDS-PAGE and immunoblotted with anti-phospho or anti-total- Akt antibody. Representative immunoblots are shown. Immunoblots were scanned to quantitate the density of the bands. All values are arbitrary densitometry units expressed relative to untreated control.

Finally, to ensure that the effects of red and white wine on modulation of survival signals in lung cancer cells were not unique to A549 cells we analyzed total and phosphorylated Akt levels in another adenocarcinoma NSCLC cell model, the H1299 cells. Exposure of H1299 cells to 2% red wine (W2, Pinot Noir) (Figure 8A), resulted in inhibition of Akt phosphorylation (27% of control), while the inhibition seen with 5% white wine (Riesling), (Figure 8B) was lower (60% of control). Total Akt levels were not affected by either red or white wine treatment (Figure 8A and B).

## Discussion

In contrast to the well-documented inverse relationship between moderate wine consumption and cardiovascular disease [11], and although limited evidence indicates that wine can inhibit cancer cell proliferation [12,13], the relationship between wine and cancer risk is not well established. Epidemiological evidence has revealed that while alcoholic beverages such as beer increase the risk for lung cancer, moderate consumption of wine is protective [18,19].

The three main subtypes of NSCLC, squamous cell lung carcinoma, adenocarcinoma and large cell lung carcinoma, account for the majority of lung cancer cases and are associated with poor prognosis [1]. In particular, NSCLC adenocarcinoma is diagnosed with increased frequency in both smokers and non-smokers with very poor overall treatment outcome despite aggressive cytotoxic therapies. For this reason we focused the present study on NSCLC adenocarcinoma. We found that red wine significantly inhibited proliferation of A549 human lung adenocarcinoma cells. All red wines examined here exhibited anti-proliferative effects, irrespective of variety and harvest time. Pinot Noir (Wine 2), a red wine with the highest phenolic content, caused the greatest inhibition of proliferation (Figure 1B) but its effect was not statistically different from the other red wines used. We used clonogenic survival assays in our studies because these experiments demonstrate the ability of cancer cells, not only to proliferate but to also develop surviving colonies that continue to grow and could develop new tumors in an *in vivo* setting. For that clonogenic assays are highly useful evaluators of both tumor growth and oncogenic potential. We observed a potent ability of red wines to induce a statistically significant inhibition of clonogenic survival at low concentration (0.02%) (Figure 2). At this concentration, white wine did not produce a statistically significant inhibition of clonogenic survival (Figure 6). A 25-fold increase in the concentration of red wines (from 0.02 to 0.5%) did not inhibit clonogenic survival further, than the one achieved by 0.02%, but this increase in concentration of white wine showed a significant inhibition of clonogenic survival (Figure 6). At high concentrations (2%) both red and white wines were able to abolish the majority of the capacity of lung cancer cells for clonogenic survival (Figures 2 and 6). These results demonstrate that although both red and white wines are able to inhibit lung cancer cell growth and oncogenic potential there is a difference in their potency. Our results are consistent with those of other studies. Elattar et al. [12] showed that red wine at concentrations similar to those used here, inhibited proliferation of human oral squamous carcinoma (SCC-25) cells. Similarly, red wine inhibited the proliferation of human prostate cancer cells [14]. Wallenborg et al. [13] observed A549 and other lung, colon and cervical cancer cell death with red wine (Raimat) at concentrations of 2.5 and 5%. However, in contrast to our observations they detected only minor, non-statistically significant, inhibition of lung cancer cells with 5% white wine (Chardonnay) and no inhibition of survival of colon and cervical cancer cells [13]. This study [13] did not examine the effects of red or white wine on cancer cell clonogenic potential.

Polyphenols are believed to mediate many of the health benefits of moderate wine consumption. Studies using red wine polyphenol extracts showed an inhibition of MCF-7 breast cancer cells [22], colon carcinoma [23], and mouse skin epidermal cells [24]. Earlier, we observed significant inhibition of prostate cancer cell clonogenic survival and radio-sensitization after treatment with 2.5-10 μM resveratrol [17]. In the present study resveratrol (1.1 μM) and ethanol matching the expected concentrations in red wine did not inhibit proliferation of A549 lung cancer cells indicating that resveratrol or ethanol alone do not mediate the effects of red wine in lung cancer cells. The lack of additivity or synergism between resveratrol 1.1 μM and red wine indicates that components already present in wine are sufficient to induce a significant inhibition of cell proliferation and survival. These observations are consistent with the results of Wallenborg et al. [13] who could not demonstrate a role of resveratrol in the red wine-mediated inhibition of cancer cell death [13]. It is possible that the total phenolic content, which is much higher in red wine (1378–2681 mg/L gallic acid equivalents) compared to white wine (3.41 mg/L gallic acid equivalents for Riesling wine used in this study), is involved in the overall anti-tumor action of wine. We suggest that future studies should investigate the effects of combined, clinically achievable, doses of polyphenols, such as quercetin, epicatechin and resveratrol, that we utilized in the present study, which as single agents do exhibit anti-tumor effects in high concentrations (Figure 1C).

The pigment content of red wine was suggested to be responsible for its cellular action. Wallenborg et al. [13] observed a correlation between pigment content, measured as absorbance at 520 nm wavelength, the inhibition of thioredoxin reductase (TrxR) and the cellular effects of different wines. Although wine pigment may contribute to the anti-proliferative effects of wine, the data obtained in our study do not offer strong support to this notion. Red wine did inhibit clonogenic survival significantly at low concentrations (0.02%) but at higher concentrations both red and white wine affected clonogenic survival to a similar extent (Figures 2 and 6).

It should be noted that in addition to polyphenols, wine also contains ethyl carbamate (10-15 ng/g) which is formed during fermentation. The metabolite vinyl carbamate has been previously reported to increase lung and intestinal mutagenicity in mice when given at a dose of 60 mg/kg [31]. Consumption of one glass of wine will result in an approximate total intake of 2.5-3.7 μg ethyl carbamate. This is a dose of 41–61 ng/kg (for a 60 kg human), which is approximately 1 million times less than the mutagenicity-inducing dose of vinyl carbamate used in the study by Hernadez et al. [31] and therefore may not cause any adverse health effects. However the presence of ethyl carbamate in wine must be acknowledged and future wine studies should explore this issue.

A number of groups, including our own [32], observed highly activated EGFR, Akt and Erk signaling pathways in human NSCLC tumours [8]. Importantly, the level of phosphorylation and activity of EGFR appears to correlate negatively with survival in lung cancer patients [8]. Overexpression of phospho-Akt in NSCLC tissue, has been found to be correlated with poor prognosis [7]. Brognard et al. [6] found phosphorylated Akt in 16 out of 19 NSCLC cell lines tested, indicating that Akt is constitutively active in NSCLC. A549 cells harbor an activating K-Ras mutation and highly activated K-Ras-PI3K pathways [33]. We observed high levels of Akt phosphorylation in these cells consistently, despite serum deprivation (Figure 4). Red wine caused a marked inhibition of basal Akt phosphorylation in both A549 (Figure 4) and H1299 (Figure 8) lung cancer cells. White wine, although not as potent in terms of inhibiting clonogenic survival, also inhibited Akt phosphorylation in both A549 (Figure 6) and H1299 (Figure 8) cells. These findings are important and indicate that wine is capable of inhibiting the activity of this kinase and this may relate to the inhibition of NSCLC cell growth. In agreement with our data, red wine has been shown to inhibit phosphorylation of Akt in vascular smooth muscle cells [34]. Although no other studies examined the effect of wine on Akt in cancer cells, we observed inhibition of basal Akt phosphorylation with 2.5 and 5 μM resveratrol in human PC3 and 22RV1 prostate cancer cells [17] and other studies in breast [35] and uterine [36] cancer cells showed similar results with 100 μM resveratrol.

The present study is the first to show a significant inhibition of Erk by red wine. Our data are in agreement with other studies showing a significant inhibition of Erk phosphorylation in human fibrosarcoma cells by resveratrol [24] and blockage of Erk phosphorylation, inhibition of the upstream kinases Raf and MEK, and suppression of the EGF or Ras-induced transformation of JB6Pt mouse skin epidermal cells by red wine extract [24].

A549 cells express wild type p53 and the present study is the first to show induction of p53 by red wine. In addition to total p53, phosphorylated p53 was increased indicating enhanced activity of this tumour suppressor. Other studies have shown increased p53 levels in response to resveratrol treatment. In thyroid [37], breast [38-41], colon [42] and prostate [17,43,44] cancer cells resveratrol significantly increased p53 levels. The increase in p53 levels seen by red wine in our study is comparable to that seen by 25 μM resveratrol in lung cancer cells [45]. *In vivo* studies have shown that red wine polyphenols reduced tumor growth in mice grafted with colon cancer cells and reduced the development of colon carcinomas, an effect that was associated with increased p53 expression [46]. p53 phosphorylation at Ser 15 leads to reduced interaction of p53 with its negative regulator MDM2, a ubiquitin ligase which targets p53 for ubiquitination and proteosomal degradation [47], resulting in increased p53 levels. On the other hand, activated Akt leads to phosphorylation of MDM2 leading to its translocation to the nucleus and inhibition of p53. It is possible that the increased p53 levels by red wine in the present study could be explained by inhibition of Akt activity on MDM2.

An important finding in the present study is the inhibition of EGF-stimulated Akt and Erk phosphorylation by red wine. High levels of expression and activation of EGFR are common in human cancer cells [2] and it is well documented that a large number of NSCLCs overexpress EGFR [2,32]. Furthermore, activating EGFR mutations are very frequent in NSCLC adenocarcinomas and are the target of modern therapy with EGFR inhibitors [48]. Treatment of A549 cells with the tyrosine kinase inhibitor gefitinib, used clinically, inhibits EGF-stimulated Akt phosphorylation [49]. In the present study we detected that wine can effectively suppress Akt phosphorylation in A549 and H1299 lung cancer cells (Figures 7 and 8) similar to clinically used EGFR inhibitors such as gefitinib.

Activated Akt leads to activation of the Ser/Thr kinases mTOR and p70 S6K involved in protein synthesis and proliferation [4,5,27]. It is well established that the mTOR- p70 S6K cascade is activated in many cancers and extensive effort has been placed on targeting this cascade in an attempt to inhibit cancer cell proliferation, tumorigenesis and metastasis [5,27]. Inhibitors of mTOR such as rapamycin and rapamycin derivatives [5,27] have been developed and are currently in clinical use or clinical trials. On the other hand, the biguanine and widely used anti-diabetic drug metformin can also inhibit the mTOR pathway and is actively investigated in cancer [28]. We have compared the effects of rapamycin and metformin with those of red wine on A549 cells and observed comparable inhibition of cell proliferation, indicating that wine may indeed deserve equal attention compared to other therapeutics currently studied against cancer.

## Conclusions

The work presented here shows that low doses of wine potently inhibit proliferation and clonogenic survival of human lung cancer cells through a mechanism that is independent of resveratrol and ethanol. Although both red and white wines demonstrated significant inhibition of cancer cell survival at high doses, red wine shows more potent anti-tumor activity compared to white. Wine inhibits basal Akt and Erk phosphorylation/activation, induces the tumor suppressor p53 and abolishes the phosphorylation of Akt and Erk by EGF. These results suggest that wine may have anti-cancer and chemo-preventive properties. Future studies need to investigate systematically the anti-tumor effects of wine in *in-vivo* models of lung cancer and analyze in detail the molecular mechanism of its action.

## Materials and methods

### Materials

Human A549 and H1299 NSCLC cells were purchased from American Type Culture Collection (ATCC). Cell culture (RPMI) media, fetal bovine serum (FBS), trypsin, and antibiotic were from GIBCO (Burlington, ON, Canada). Total and phospho-specific Akt, Erk and p53 antibodies were purchased from New England Biolabs (Mississauga, ON, Canada). [^3^H]thymidine and [^3^H]2-deoxy-D-glucose were from PerkinElmer (Boston, MA). Metformin, rapamycin, resveratrol, quercetin, catechin, epicatechin, bovine serum albumin and other chemicals were purchased from Sigma (Oakville, ON). Red and white wine was prepared by Dr Pickering at the Cool Climate Oenology and Viticulture Institute (CCOVI), Brock University. The grapes used were from vineyards in Niagara-On-The-Lake, Ontario. The basic physicochemical composition and phenolic content of the wines were measured as previously described [50,51].

### Cell culture and treatment

A549 and H1299 cells were grown in RPMI media containing 5 mM glucose, 10% (v/v) FBS, and 1% (v/v) antibiotic-antimycotic solution (100 U/ml penicillin, 100 μg/ml streptomycin, and 250 ng/ml amphotericin B) in a humidified atmosphere of 5% CO_2_– 95% air at 37°C. The final concentration and the time of incubation with wine or other agents are indicated in each figure. Where indicated the cells were stimulated with 100 ng/ml EGF for 10 min.

### Thymidine incorporation

Subconfluent A549 cells were serum-deprived for 24 hours and then treated with the indicated concentrations of wine (added to media) for 12 hours followed by addition of 10 μM [^3^H]thymidine for an additional 12 hours. At the end of the treatment, the media was removed, the cells were rinsed with ice-cold HEPES-buffered saline (HBS; 140 mM NaCl, 5 mM KCl, 20 mM HEPES, 2.5 mM MgSO_4_, and 1 mM CaCl_2_, pH 7.4) and subsequently, the unincorporated [^3^H]thymidine was precipitated out of the cells with 10% trichloro- acetic acid (TCA) for 10 minutes at 4°C. The TCA was aspirated and the cells were rinsed twice with ice-cold HBS, followed by solubilization with 0.05 N NaOH and radioactivity counting. Cellular protein content was measured using the BioRad Protein Assay.

### Clonogenic assays

Clonogenic assays were performed as described previously [17]. Cells (500–1000) were seeded in triplicates, allowed to adhere overnight and were incubated with media containing the indicated concentrations of red wine, resveratrol, or ethanol (to match the ethanol in wine-treated cells) for 7 days. Cells were then fixed and stained with 0.05% methylene blue and colonies (> 50 cells) were counted. Results are expressed as the surviving fraction compared to untreated control.

### 2-Deoxy-D-glucose uptake

Glucose uptake measurements were carried out in HBS containing 10 μM 2-[^3^H]-Deoxy-D-glucose as previously described [52]. The uptake assay was terminated by washing the cells with ice-cold 0.9% NaCl, followed by cell solubilization with 0.05 N NaOH and radioactivity counting.

### Immunoblotting

After treatment, cell lysates were prepared followed by separation of 20 μg of protein sample by sodium dodecyl sulfate-polyacrylamide gel electrophoresis (SDS-PAGE) and immunoblotting with the indicated antibodies as described earlier [53]. Densitometric analysis was performed using Image J software.

### Statistical analysis

The results are the Mean ± SEM of the indicated number of independent experiments. Analysis of variance (ANOVA) or student’s paired t-test was used. Statistical significance was assumed at P < 0.05. SPSS v19.0 was used.

## Competing interests

The authors declare that they have no competing interests.

## Authors’ contributions

CCB performed most experiments and contributed to the manuscript preparation. JM performed some experiments, contributed to data analysis and figure preparation. GP produced all the wines and performed the wine analysis. TT contributed to the design of the study, data interpretation, data presentation and manuscript preparation. ET was responsible for the conception and design of the study, data analysis, data interpretation, data presentation and manuscript preparation. All authors read and approved the final manuscript.
